# The Effect of Age, Gender, and Insertion Site on Marginal Bone Loss around Endosseous Implants: Results from a 3-Year Trial with Premium Implant System

**DOI:** 10.1155/2014/369051

**Published:** 2014-08-12

**Authors:** Massimiliano Negri, Carlo Galli, Arianna Smerieri, Guido M. Macaluso, Edoardo Manfredi, Giulia Ghiacci, Andrea Toffoli, Mauro Bonanini, Simone Lumetti

**Affiliations:** ^1^Private Practice, 29121 Piacenza, Italy; ^2^SBiBiT, Centro di Odontoiatria, Università degli Studi di Parma, Via Gramsci 14, 43126 Parma, Italy

## Abstract

*Objectives.* The goal of this study was to evaluate bone changes around endosseous implants in partially edentulous patients. *Materials and Methods.* A total of 632 two-stage implants were placed in 252 patients. The implants had straight emergence profile, ZirTi surface, 3.3 to 5 mm diameter, and 8.5 to 13 mm length. Bone levels were assessed on orthopantomography immediately after surgery and after 36 months and marginal bone loss (MBL) was calculated from their difference. *Results.* Cumulative survival rate was 98.73%. Overall MBL was 0.8 mm ± 0.03 (mean ± SEM). Higher MBL was observed around implants in the maxilla than in the mandible (*P* < 0.007). A relation between implant diameter and MBL (*P* < 0.0001) was observed in male and, more limitedly, female patients. Older patients had higher MBL in the maxilla, but not in the mandible (*P* < 0.0001). MBL progressively increased with age in male patients, but reached a peak already in the 50–60 years age group in the female subset (*P* < 0.001).* Conclusions.* The overall MBL is consistent with the available literature. Site difference and patient age and gender appear to significantly affect MBL, representing important factors to be considered during implant placement.

## 1. Introduction

Stability of peri-implant tissues is considered crucial when evaluating dental implant outcomes [1, 2] and it is measured by clinical and radiologic parameters. Marginal bone loss (MBL) is one of the most important of them because bone around implants is necessary for mechanical stability and plays a key role in esthetic outcomes as well [3] as the presence of adequate levels of bone around implants directly affects soft tissues and, as direct consequence, esthetics and hygienic maintenance.

Limited resorption around implants is generally considered normal [4, 5] and most reports agree that MBL should not exceed 1 mm at 1 year after prosthesis positioning [6–9], although values up to 2 mm have been reported [10]. A recent review [8] found that few implant systems have any published data about MBL and concluded that this piece of information should be available in the scientific literature for all dental implant systems on the market. Marketing strategies often employ implant performance data derived from studies on implant designs that have been discontinued or modified throughout the years. As a matter of fact, most of the dental implant systems used worldwide do not provide adequate outcome data, and this is particularly apparent when long term results are sought.

The aim of this longitudinal study was to evaluate the effect that patient age and gender, implant diameter, and insertion site have on marginal bone loss around implants in partially edentulous patients. We report on the 3 years results.

## 2. Materials and Methods

This study is a longitudinal analysis of a consecutive series of partially edentulous patients who underwent implant-supported fixed rehabilitation. Treatments were performed in the year 2009 by the same dentist, in a private practice setting.

### 2.1. Patient Enrollment

Consecutive patients of a private dental practice during a one-year time span were considered eligible.

*Inclusion Criteria*. Patients had to be at least 18 at the start of the study; had to present with partial edentulism, adequate oral hygiene, that is, plaque index score ≤10%. If oral hygiene was inadequate, it was improved until patients were within the set criteria.

*Exclusion Criteria*. Patients were excluded from the study if they presented with any of the following conditions: a history of leucocyte dysfunction; history of bleeding disorders; history of renal failure; patients with metabolic bone disorders; patients with uncontrolled endocrine disorders; alcoholism or drug abuse; HIV infection; smoking >25 cigarettes a day or cigar equivalents.

Two hundred forty-seven partially edentulous patients requiring single or multiple implants and single crown or bridge prosthetic rehabilitation were included in the study. Before enrollment, all patients received written and verbal information and gave their written consent to the treatment plan.

### 2.2. Surgical and Restorative Procedures

Before surgery, impressions and bite registration were taken and an ideal prosthetic setup of the tooth/teeth to be restored was conducted.

All patients received antibiotics prophylaxis with 2 g of amoxicillin 1 hour before surgery. After local anesthesia (articaine with adrenaline 1 : 100000), mucoperiosteal flaps were raised extending the incisions to the adjacent teeth. Implant osteotomies were prepared with progressive-diameter burs under abundant saline irrigation, according to the manufacturer's instructions. Surgical templates were used to facilitate optimal prosthetic implant positioning. All implants were premium implants with straight emergence profile and ZirTi surface (zirconium sandblasted acid etched titanium, Sweden & Martina, Due Carrare, Padova, Italy). Implant diameter ranged from 3.30 to 5 mm and length from 7 to 13 mm. Implant diameter was chosen based on the site to rehabilitate.

Implants were placed at minimum distance of 1,5 mm from the adjacent teeth and at 3 mm interimplant distance. Implant shoulders were always placed at crest level, except when an immediate postextraction implant insertion was performed. In this case, the implant was placed 1 mm deeper than the vestibular osseous margin. If a gap was present between implant and alveolar bone walls, DBBM (deproteinized bovine bone mineral, Bio-Oss, Geistlich, Wolhusen, Switzerland) was employed.

Antibiotics (amoxicillin, 1 g 3 times a day for 6 days) and 0.2% chlorhexidine rinses (2/day for 15 days) were prescribed after surgery. After a healing period ranging from 3 to 4 months, implants were uncovered and a temporary acrylic prosthesis was placed on custom-made or pre-formed abutments. After approximately 2 months, porcelain-metal or porcelain zirconia prosthesis were connected to the abutments.

### 2.3. Recall Visits

Follow-up visits were scheduled at 6 months for clinical reevaluation. Oral hygiene was monitored and further instructions were given as needed in order to keep the plaque index below 10%. Patients were monitored at 6 month recall visits if no mucositis was present; otherwise, they received hygiene instructions and professional hygiene and were visited again after a month. The condition of the gingiva around all teeth present and the mucosa around the implants was assessed at four surfaces (mesial, distal, buccal, and palatal) using a periodontal probe. Inflammation in the soft tissue was considered present when the site bled on gentle probing. Assessments were made at all visits. The proportion of sites that showed the presence of inflammation (full-mouth bleeding score, FMBS) was calculated.

Orthopantomographies were taken at 6 and 36 months for subsequent analysis of marginal bone loss (MBL).

### 2.4. Clinical Outcome

Implant stability was clinically evaluated at every follow-up visit and the occurrence of complications including pain, infection, mucosal dehiscences, peri-implant mucositis, and prostheses/abutments break/unscrewing were also recorded. Implant cumulative survival rate (CSR) was calculated at 36 months.

### 2.5. Radiographic Outcome

**Figure 1:**
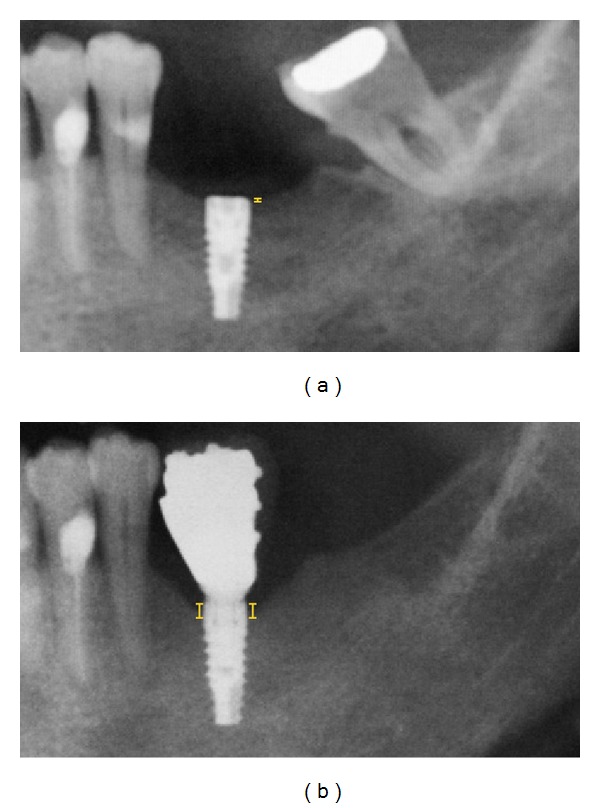
Detail of orthopantomographies 6 months (a) or 3 years (b) after implant insertion. MBL was calculated from the distance between implant shoulder and first bone-to-implant contact, which here is indicated by a yellow solid line.

Radiographic assessments of implants at 6 and 36 months were performed using OPTs. The radiographic equipment was regularly calibrated according to the manufacturer's instructions. OPT images were saved as digital imaging and communications in medicine (DICOM) files. Each DICOM file was subsequently downloaded to a personal computer. An image analysis software (OsiriX Imaging Software, an open source freeware for academic use from the OsiriX webpage (http://www.osirix-viewer.com/) was used to measure peri-implant MBL along the vertical plane, as the distance between implant shoulder and first bone-to-implant contact (Figure 1). Marginal bone loss was calculated as the difference between MBLs at 6 and 36 months. To ensure a standardized comparison, no adjustments to brightness and contrast settings were allowed. Two calibrated blinded examiners (Pearson correlation *r* ^2^ = 0.75) performed all the readings and mean values were used for subsequent analysis.

### 2.6. Statistics

Standard statistical analysis was performed using the statistical package SPSS 18.0. The normal distribution of age was analyzed and the population was divided into 3 age groups: group 1 (<50 yrs), group 2 (51–60 yrs), and group 3 (>61 yrs).

The relationship between MBL and age was determined by Pearson correlation both in the general population and in male or female patients.

Data were presented as mean ± SEM. Dropouts and failed implants were not included in the analysis. Data normality was verified using Kolmogorov-Smirnov, *U*-Mann Whitney tests, and descriptive analysis. Data were analyzed by general linear model (GLM) which subsumes traditional regression, ANOVA, and ANCOVA including repeated measures analysis.

The analysis was performed to identify the categorical variables (type of implant, bone localization, gender, and age) significantly associated with MBL. A *P* value < 0.0125 was considered statistically significant, based on Bonferroni correction for 4 variables.

## 3. Results

**Figure 2:**
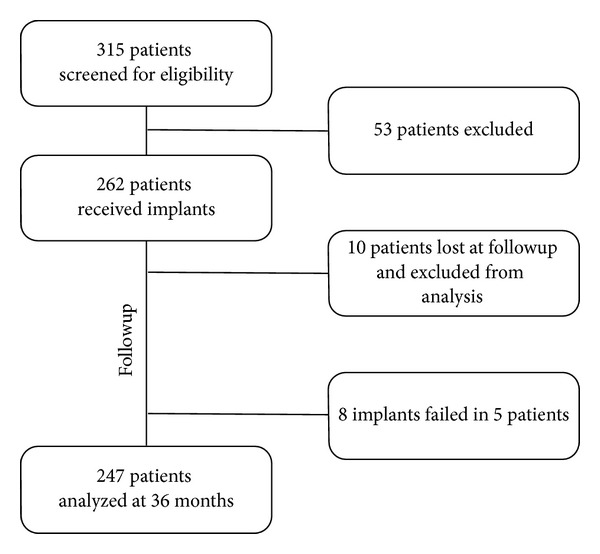
Flow chart illustrating the total number of patients screened, enrolled, and analyzed in the study.

A total of 315 patients were screened for eligibility and 53 patients were excluded on the base of the inclusion and exclusion criteria (Figure 2). Two hundred sixty-two patients were enrolled in the study, ten patients were lost at followup and were not included in the analysis. Eight implants in five patients failed in the course of the study, namely, three 3.80 mm implants, four 4.25 implants, and one 5 mm implant (cumulative survival rate = 98.73%). All the failed implants were successfully replaced, and the patients were considered as dropouts for the MBL analysis.

The remaining 247 patients, 103 male and 144 female, received 624 implants. The mean age of the analysed population is reported in Table 1. Implant distribution by bone type and gender is reported in Table 2.

### 3.1. The Effect of Implant Platform and Insertion Site on MBL

**Figure 3:**
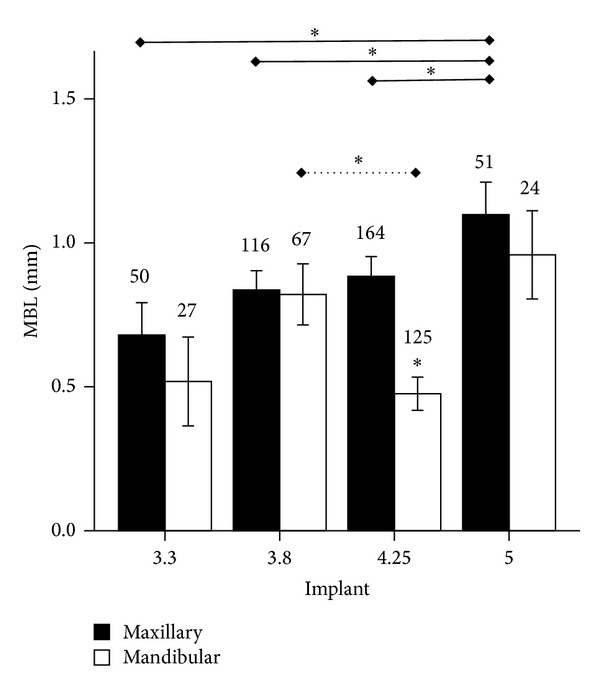
Marginal bone loss (MBL, mm) around endosseous implants of different diameter (3.3, 3.8, 4.25, or 5 mm) inserted in maxilla or mandible. *P < 0.05. Solid line indicates comparisons in the maxilla; dotted line indicates comparisons in the mandible.

Regardless of patient gender and age, higher MBL was observed around implants inserted in the maxilla as compared to the mandible, and this difference was statistically significant for the 4.5 mm platform (*P* < 0.0001) (Figure 3).

A relation between implant diameter and MBL was observed by Pearson analysis (*P* = 0.046). When the maxilla alone was considered, lower MBL around 3.3 mm implants and higher MBL around 5 mm implants were recorded (0.7 ± 0.1 versus 1.1 ± 0.1; *P* < 0.0001) (Figure 3).

**Figure 4:**
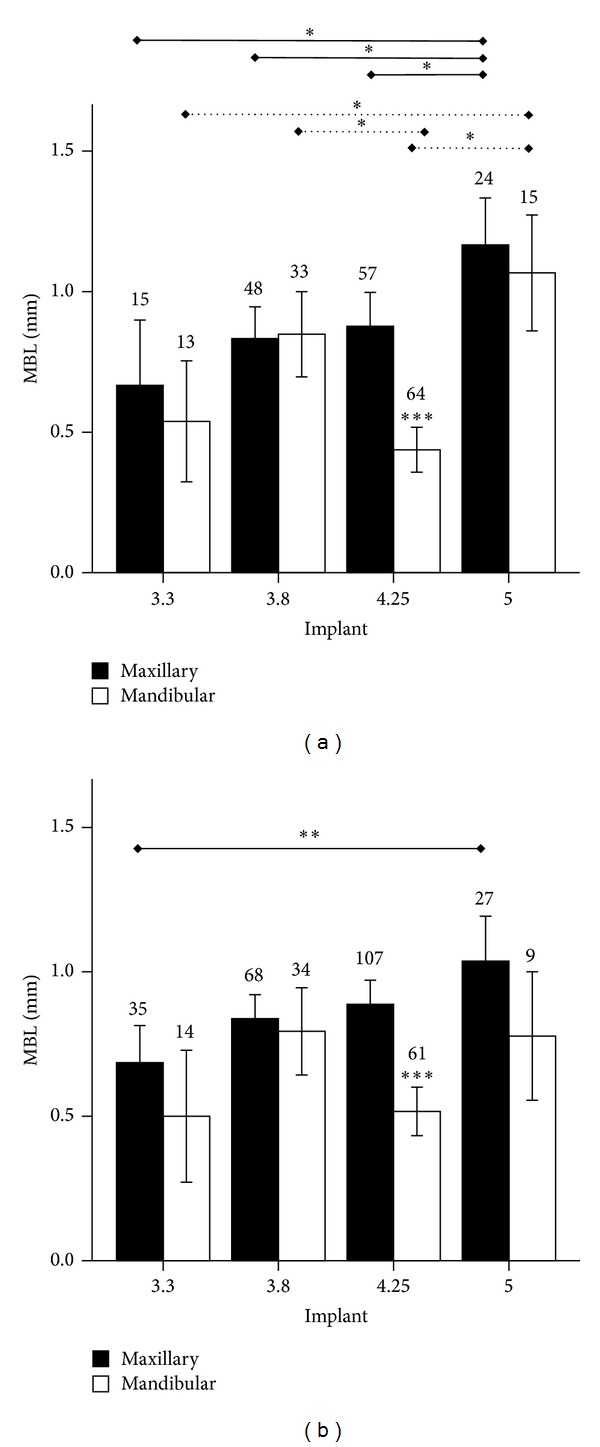
Marginal bone loss (MBL, mm) around endosseous implants of different diameter (3.3, 3.8, 4.25, or 5 mm) inserted in maxilla or mandible in (a) male or (b) female patients. *P < 0.05, **P < 0.01, and ***P < 0.0001. Solid line indicates comparisons in the maxilla; dotted line indicates comparisons in the mandible.

When patients were stratified by gender, a relation, albeit not statistically significant, between MBL and implant platform in maxilla was observed both in male and female patients. A similar trend was observed in the mandible as well, with the only exception of 4.25 mm implants (Figures 4(a) and 4(b)).

### 3.2. The Effect of Age on MBL

Pearson analysis indicated a correlation between patient age and MBL (*P* = 0.03). When maxilla alone was considered, a correlation between MBL and age was observed (*P* = 0.001), though no such correlation existed in the mandible. A correlation was also observed both in the maxilla (*P* = 0.01) and in the mandible (*P* = 0.048) in male subjects, whilst only in the maxilla in the female group (*P* = 0.02).

**Figure 5:**
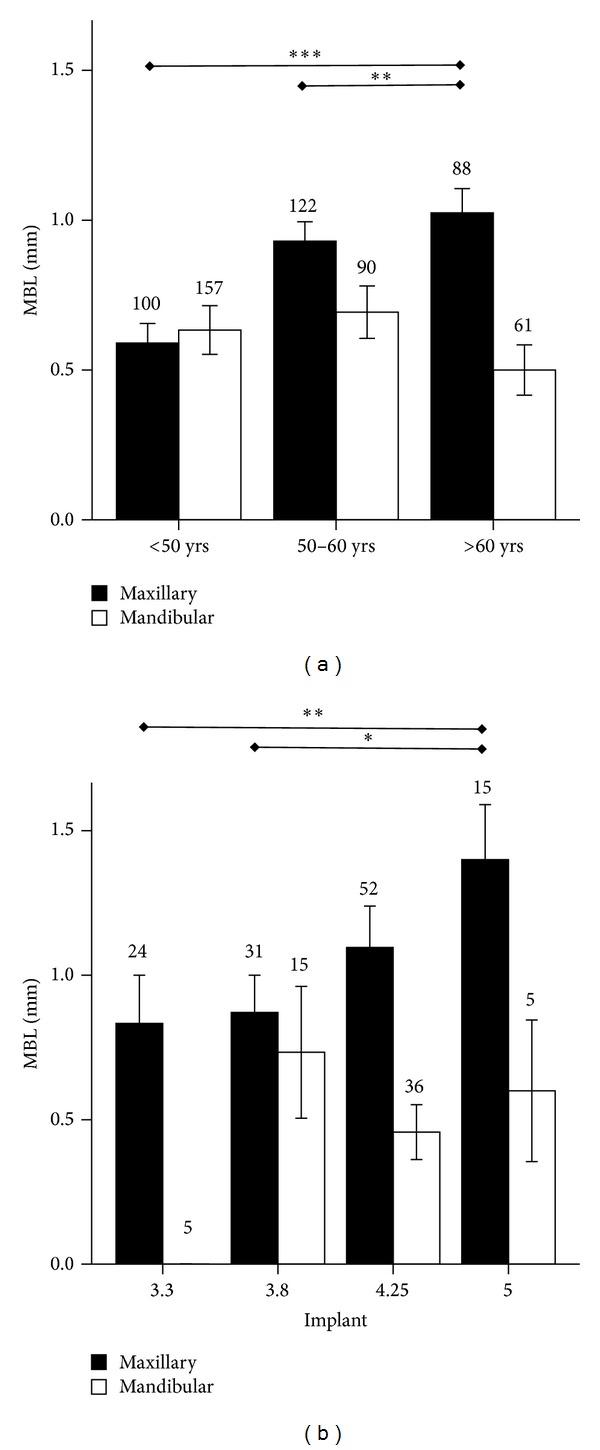
(a) Marginal bone loss (MBL, mm) around endosseous implants inserted in maxilla or mandible in patients of different age categories. (b) Marginal bone loss (MBL, mm) around endosseous implants of different diameter (3.3, 3.8, 4.25, or 5 mm) inserted in maxilla or mandible in patients older than 60 years. *P < 0.05, **P < 0.01, and ***P < 0.0001.

**Figure 6:**
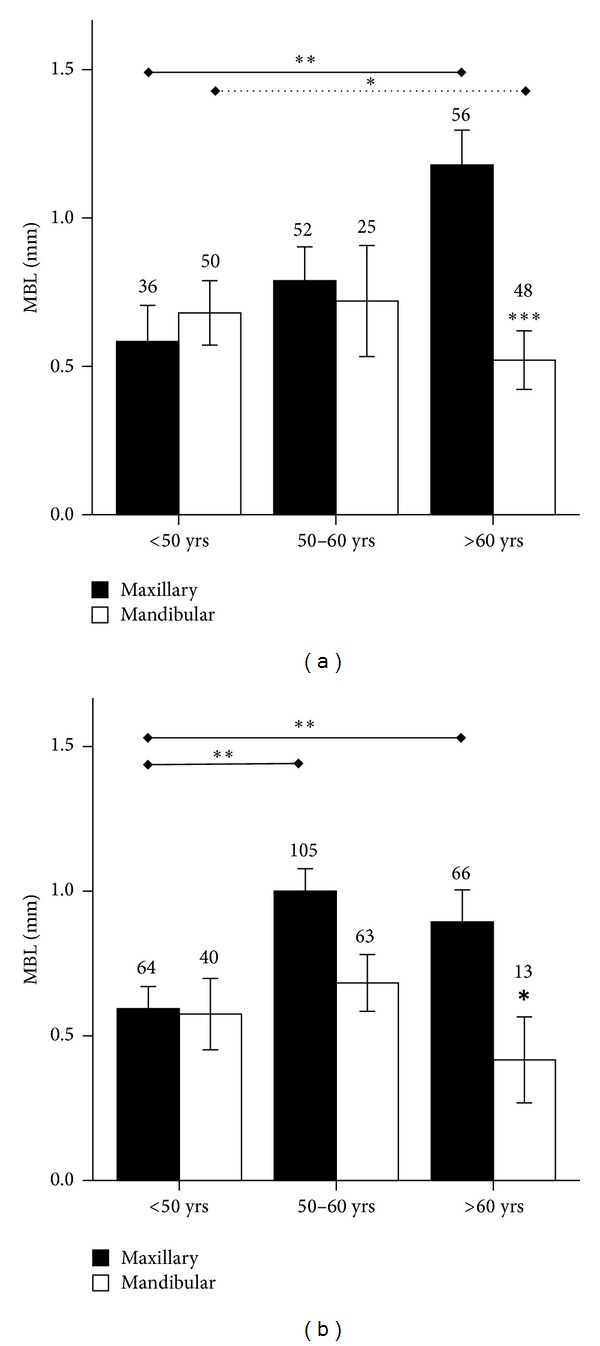
Marginal bone loss (MBL, mm) around endosseous implants inserted in maxilla or mandible in (a) male or (b) female patients of different age categories. *P < 0.05, **P < 0.01, and ***P < 0.0001. Solid line indicates comparisons in the maxilla; dotted line indicates comparisons in the mandible.

Based on age distribution in the study population, three age groups were identified: group (1) <50 years old, group (2) 50–60 years old, and group (3) >60 years old. Older patients had progressively higher MBL in the maxilla (Figure 5(a)), but this did not occur in the mandible (*P* < 0.03), where no difference across age groups was observed. A relation between implant platform and MBL was observed in group 3 but not in groups 1 and 2 (Figure 5(b)). When the effect of gender was taken into account, MBL appeared to progressively increase with age in male patients and reach a peak in group 3, though MBL reached a plateau already in group 2 in female patients and no difference between group 2 and 3 was observed in this patient subset (*P* < 0.001) (Figures 6(a) and 6(b)).

### 3.3. Prosthetic Problems and Other Complications

Chipping of 6 crowns or bridges in 5 patients was found at followup. Four single-tooth restorations presented with abutment loosening (data not shown).

## 4. Discussion

The purpose of the present study was to evaluate the effects of insertion site, platform, age, and gender on marginal bone resorption around endosseous implants over a period of 3 years, when used to support fixed prosthesis in the maxilla or in the mandible. Marginal bone loss (MBL) around implants is an important parameter for implant success and soft tissue esthetics and is known to be significantly affected by implant design. Bone is more resistant to compressive forces than to shear forces, and it is particularly sensitive to lateral forces exerted around the implant collar, where marginal resorption occurs [11]. Thread design, spacing, and implant diameter can all affect force transfer to bone [12], and therefore every new implant system should be tested to investigate its effectiveness to preserve adequate bone levels. MBL is affected by surgical technique to a lesser extent [13], and no differences were reported in the literature between one- or two-stage implant placement, in the absence of infection [14].

The placement of immediate postextractive implants is generally considered as predictable as their delayed placement in terms of implant success, survival, and complications, although few RCT studies are available [15].

The first three years of implant use are decisive for MBL [16], and it has been shown that most resorption occurs during the first year after surgery and this process slows down during the second year and stabilises to an average 0.05–0.15 mm/year bone loss rate [6, 8, 17]. The present study showed a 98.7% success rate after 3 years, with an average MBL of 0.9 ± 0.1 mm in the maxilla and 0.7 ± 0.2 mm in the mandible, a consistent behaviour within the accepted limits for MBL according to the current literature [6–9], although longer followups will be needed for decisive conclusions. The overall success rate was well within the limits indicated by the literature, and with the only exception of one implant, for which no plausible clinical explanation could be adduced, failures were limited to implants in sites treated with guided bone regeneration procedures. Although standardized intraoral radiographs are a more precise method to visualize peri-implant bone changes over time, panoramic radiographs have often been used in the existing literature [18, 19] and were used in the present study to control anatomical structures such as the maxillary sinus, mental foramen, mandibular canal, and adjacent teeth, and additional exposure to ionising radiations for the purpose of the study was deemed ethically unacceptable. However, to minimise bias, the OPT machine that was used for all the patients was regularly calibrated, and its distortion was independently determined to be below 15%. Although this did introduce a bias in our measurements, given its magnitude, we do not believe this significantly affected them, as no differences below 1 mm were directly measured from the OPTs.

Implants placed in the mandible tended to have smaller MBL than in the maxilla after 3 years. Tissue architecture accounts for a likely candidate for this difference. The denser mandibular bone can more effectively withstand loading while undergoing slower remodelling around the bone collar than the maxilla, which is richer in cancellous bone. When considered separately, smaller platforms displayed a tendency to a reduced MBL as compared to wider platforms in the maxilla in both genders and, when male patients were considered, in the mandible as well. Possible causes for the observed discrepancies can only be object of speculation and be related to biomechanical differences in loading intensity among different sites. A larger diameter requires the implant to be inserted in more posteriors regions of the ridge, where tissue architecture is different and mechanical loads are higher. Larger implants are therefore expected to be subject to higher compressive forces and these may have caused more resorption. Prosthetic factors can also account for at least part of the differences in bone resorption, as it has been shown that switching to a narrower platform when applying the abutment can help preserve marginal bone. No platform switching was performed with the implants used in the present study.

We stratified patients into three age groups: <50 years old, 50 to 60 years old, or >60 years old. No difference in MBL was observed across the three age groups around implants placed in the mandible, whilst higher MBL was recorded around implants in the maxilla in older patients. Age is an important factor for bone maintenance, as it is known that bone mass density decreases with aging, as it occurs with age-related osteoporosis in male and female patients. It is also known that age-related bone loss is predominant in the cancellous compartment because its underlying mechanisms, such as increased oxidative stress, directly control osteoclast activity on trabecular bone but more limitedly affect cortical bone [20, 21].

Moreover, interestingly the peak of MBL was reached in the 50- to 60-year-old age group in the female patient subset, and no further increase in MBL was seen in the >60 group. This could be correlated with menopause onset, a complex series of physiologic events that normally take place around that age and that can have a profound impact on bone resorption as well, while sharing similar pathogenetic mechanisms with age and possibly synergizing, and overriding age effects on bone mass [22].

## 5. Conclusions

Taken together, this data indicates that significant differences exist in the marginal bone loss around implants inserted in the different sites, with different diameter and in patients of different age and gender. Larger implants in the maxilla have higher MBL than smaller platforms and older patients have higher MBL when they receive implants in the maxilla, while the mandible yields more consistent results across platform and regardless of patient age. Therefore, within the accuracy limits of the present study due to the use of OPTs and a 3-year followup, this data suggest that implant and patient characteristics should be considered when placing an implant in the maxilla, because they can significantly affect the clinical behaviour of bone around the implants.

## Figures and Tables

**Table 1:** Mean age of analyzed patients.

	Total (number)	Male (number)	Female (number)
Total patients	54.4 ± 0.7 (247)	55.8 ± 1.1 (103)	53.3 ± 0.8 (144)
Maxilla	55.2 ± 0.9 (137)	56.9 ± 1.6 (49)	54.3 ± 1.0 (88)
Mandible	53.2 ± 1.0 (111)	54.8 ± 1.7 (54)	51.6 ± 1.0 (57)

Mean ± SEM (number of patients).

**Table 2:** Number of implants.

	Maxilla (M/F)	Mandible (M/F)	Total (M/F)
Diameter 3.3 mm	50 (15/35)	27 (13/14)	77 (28/49)
Diameter 3.8 mm	116 (48/68)	67 (33/34)	183 (81/102)
Diameter 4.25 mm	164 (57/107)	125 (64/61)	289 (121/168)
Diameter 5 mm	51 (24/27)	24 (15/9)	75 (39/36)

Total	381 (144/237)	243 (125/118)	624 (269/355)
